# Long Chain Polyunsaturated Fatty Acids (LCPUFAs) in the Prevention of Food Allergy

**DOI:** 10.3389/fimmu.2019.01118

**Published:** 2019-05-22

**Authors:** Tamara Hoppenbrouwers, Jelena H. Cvejić Hogervorst, Johan Garssen, Harry J. Wichers, Linette E. M. Willemsen

**Affiliations:** ^1^Food Quality and Design, Wageningen University & Research, Wageningen, Netherlands; ^2^Department of Pharmacy, Faculty of Medicine, University of Novi Sad, Novi Sad, Serbia; ^3^Department of Immunology, Nutricia Research BV, Utrecht, Netherlands; ^4^Division of Pharmacology, Department of Pharmaceutical Sciences, Faculty of Science, Utrecht University, Utrecht, Netherlands; ^5^Food & Biobased Research, Wageningen University & Research, Wageningen, Netherlands

**Keywords:** food allergy, PUFA, LCPUFA, immune response, anti-inflammatory

## Abstract

N-3 long chain polyunsaturated fatty acids (LCPUFAs) are considered to possess protective properties for human health by impacting on immunological reactions. An “inflammation-suppressive” effect appears to be the common denominator of the beneficial effects of most of these dietary components which may protect against the development of chronic immune disorders such as (food) allergy. LCPUFAs, especially n-3 LCPUFAs, have been shown to interact with both the sensitization as well as the effector phase in food allergy in pre-clinical models. In this review, we explore the anti-allergic properties of LCPUFAs by providing an overview of clinical, *in vivo* and *in vitro* studies. Furthermore, we discuss the susceptibility of LCPUFAs to lipid oxidation and possible strategies to support the efficacy of LCPUFAs in reducing the allergy risk by using additional components with anti-oxidative and anti-inflammatory capacities such as the flavonoid quercetin. Finally, we propose new strategies to prevent (food) allergy using combinations of LCPUFAs and additional nutrients in diets or supplements, and postulate to investigate the use of LCPUFAs in allergic symptom relief.

## Introduction

Allergic reactions, particularly as a result of food allergy, can be life-threatening. The frequency of reported allergies and the severity of allergies in the Western world has increased significantly, and is forecasted to affect e.g., 50% of the EU's population by 2025 (1). Food allergy is one of the first allergies to arise early in life and has an overall worldwide increasing prevalence of 5–10%, highly dependent on the country (2, 3). The majority of these allergies are triggered by milk, eggs, peanuts, other nuts, wheat, soy, and (shell)fish. Of these, reactions to milk, eggs, and peanuts are the most prevalent in children, while peanuts and (shell)fish are the major triggers of allergic reactions in teenagers and adults (4).

The majority of food allergy is known as a type I allergy, indicating that it is mediated by a relatively acute response in which immunoglobulin E (IgE) is the pivotal antibody involved. However, also in the absence of allergen specific IgE, acute allergic responses may occur upon ingestion of the culprit food allergens. The immunological mechanisms behind food allergy have been extensively explained previously (5). Briefly, food allergy can be subdivided into two phases: allergic sensitization and the allergic effector response.

### Allergic Sensitization

Allergenic proteins in foods, are taken up by antigen-presenting cells (APCs), and of these, dendritic cells (DCs) are mainly involved in the presentation of antigens to naive T-cells. This normally results in tolerance toward harmless food proteins. However, Th2 polarized immunity and mixed Th2 and Th1 driven allergen specific immune responses occur in the acute and chronic phase in allergy, respectively. In order to activate T-cells, two signals are needed: the binding of the T-cell receptor (TCR) specifically recognizing the allergenic epitopes presented via MHCII by DCs, and interaction of the co-stimulatory molecules CD28, CTLA-1/4 and LFA-1, and CD134 on the T-cell with, respectively, B-7 (CD80/CD86) and ICAM-1 and OX40L on the DC (6). Differentiation into CD4+ T-cells, or T-helper (Th) cells Th1, Th2, and Treg is regulated by many different factors, e.g., cytokines and/or chemokines such as IL-12 and IFNγ (Th1), IL-4, CCL17, and CCL22 (Th2) and IL-10 (Treg) and co-stimulatory molecules such as LFA-1/ICAM-1 (Th1) and CD134 and OX40L (Th2) (7). In allergy, OX40L expression by DCs has been reported to be the most important Th2 driving factor (8). Th2 cell delivered IL-4 plays a key role in development of type 1 allergy by driving IgE secretion via induction of allergen specific IgE isotype switching in B-cells and antibody production by plasma cells, resulting in allergic sensitization (9, 10). Beyond binding to the FcεRI of allergic effector cells such as mast cells and basophils, IgE can also bind to this receptor on DCs, further stimulating the immune response. Finally, Th1 cell derived mediators (IFNγ) downregulate Th2 cell proliferation and Treg cells are able to downregulate the proliferation and activation of both Th1 and Th2 cells.

### Allergic Effector Response

Upon a second encounter with the allergen, crosslinking of the IgE bound FcεR1 receptor by the allergen results in mast cell and basophil activation and degranulation and the induction of the allergic effector response. Mast cells release many different components such as histamine, proteases, heparin, leukotrienes, prostaglandins, cytokines, and chemokines, which are all involved in the generation of the allergic symptoms by causing redness, swelling, and vasodilation. Sometimes this may even lead to an anaphylactic highly acute reaction within minutes.

### Dietary and Environmental Determinants of Food Allergy Risk

The development of the gastro-intestinal and systemic immune system is in part driven by the intestinal microbiome. Microbiome disturbances have been reported to be associated with different types of allergies (11). Changes in (children's) microbiome are claimed to be important in the increase in food allergy cases although it is not completely evident what is cause or consequence (12). Such microbiome changes can be caused by, among others, an increased intake of fat and processed food, reduced intake of dietary fibers, fruits and vegetables, and the use of antibiotics during pregnancy and/or in early life (12–14). Currently no standard treatment is available for food allergy, therefore, the need for prevention and resolving allergy is becoming of major concern. One of the factors that may contribute to the risk of developing food allergy is the quantity and quality of fat used in current diets. The typical Western diet is rich in n-6 polyunsaturated fatty acids (PUFAs) and poor in n-3 PUFAs. The ideal balance of n-3:n-6 PUFAs has been established to be between 1:3 and 1:5. However, in the current Western diet, the balance has been shifted to 1:10 to 1:30, which is dramatically out of balance at the expense of the n-3 PUFAs. This shift can affect both the microbiome (15) and the immune system (16) of unborn children when the mother consumes food rich in n-6 PUFA. It has been recognized that nutrition plays an important role in the development, maintenance, and appropriate functioning of the immune system, and consequently it may also contribute to the prevention and management of for example food allergies. Food constituents, such as long-chain polyunsaturated fatty acids (LCPUFAs) may be able to influence the allergic sensitization and/or effector response through multiple biological pathways. In this review we will explore the current knowledge on the use of LCPUFAs in the prevention of food allergy, and aim to provide insights to improve future outcomes.

## Long-chain Polyunsaturated Fatty Acids

**Figure 1:**
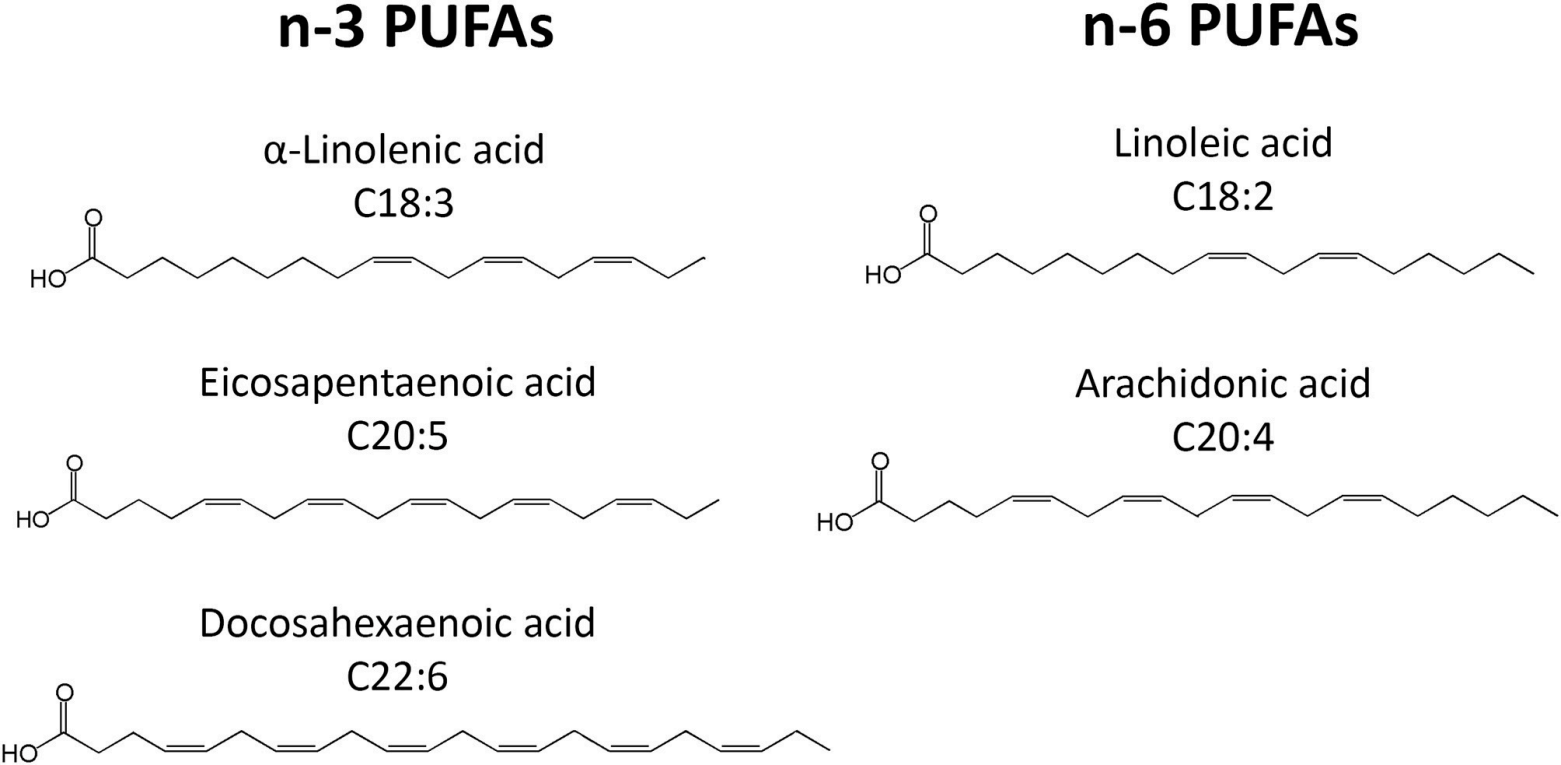
Schematic overview of the chemical structures of the n-3 and n-6 PUFAs discussed here.

PUFAs are a group of acids that contain more than one double bond in their molecular structure. The most important PUFA groups are omega-3 (n-3) and omega-6 (n-6), depending on the placement of the first double bond, which is either at the third or the sixth carbon of the methyl end (Figure 1). In the n-3 group, essential α-linolenic acid (ALA, 18:3n-3) is enzymatically converted into stearidonic acid (SDA), and elongated into long-chain eicosapentaenoic acid (EPA, 20:5n-3), which is converted into docosapentaenoic acid (DPA, 22:5n-3) and then docosahexaenoic acid (DHA, 22:6n-3). In the n-6 group, essential linoleic acid (LA, 18:2n-6) is converted into long-chain arachidonic acid (AA, 20:4n-6) (Figure 1). However, the conversion rate to LCPUFA is limited, and n-3 PUFA compete with n-6 PUFA for conversion since the same elongation and desaturation enzymes are used. The n-3 LCPUFA can be obtained from fatty fish, such as salmon, tuna, mackerel, herring and sardines, and fish oil. More sustainable sources such as vegetable oil, nuts, and seeds contain n-3 PUFA ALA, while algae oil is rich in n-3 LCPUFAs DHA and EPA.

As the result of dietary changes over the last decades, the balance between n-3 and n-6 PUFAs has been disturbed in favor of n-6. N-6 PUFAs, present in vegetable oils, such as sunflower, soybean, and corn oil, are increasingly consumed, while the intake of n-3 LCPUFA, at least in westernized countries, is generally low. Since n-6 LCPUFA AA is associated with pro-inflammatory and n-3 LCPUFAs EPA and DHA with anti-inflammatory activities, the mentioned imbalance is possibly contributing to the rise of non-communicable diseases, including allergies. Usually recommended consumption of two portions of fatty fish per week corresponds to 200 mg DHA per day (17). Due to efficient digestion and absorption, approximately more than 95% of ingested fatty acids become biologically available and get incorporated in the phospholipid bilayer of cell membranes (18).

## Immunomodulation by PUFAs in Food Allergy

**Figure 2:**
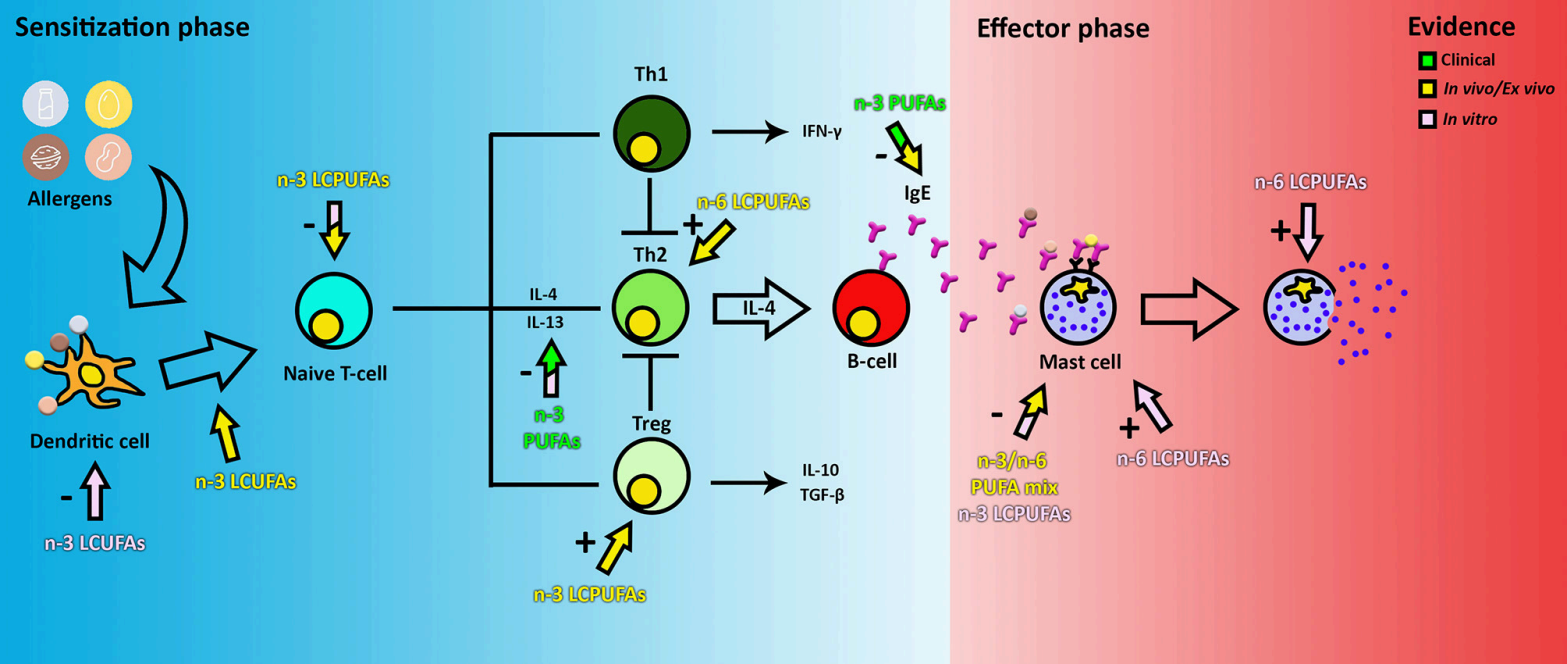
The effect of PUFAs on food allergy. The color of the arrows and text indicate if the evidence is obtained from clinical, *in vivo* or *in vitro* data. The + or—indicates if the observed effect is an inhibitory or stimulatory response of a certain cell type. Note that clinical and *in vivo* arrows indicate the observed end stage effects only, this may not be a reflection of the direct effect of PUFAs on the target cells. Therefore, the components could actually target a cell group earlier in the pathway.

PUFAs have attracted attention for prevention of food allergy for many years, mainly because they are able to target nearly every cell type within both the allergic sensitization and effector phase (Figure 2). Upon ingestion, PUFAs are incorporated into the cell membrane, thereby influencing cell properties such as membrane fluidity and the inflammatory response (19). In *in vitro* experiments, PUFAs are usually added in concentrations ranging from 2 to 100 μM (20–23) to study the effect on mast cells, DCs, T-cells, or a combination of the latter two. Despite this variation, most studies report similar results.

During the sensitization phase, PUFAs are able to intervene in pathways of DCs, T-cells, and IgE production by B-cells as shown in pre-clinical models. In mouse myeloid DCs activated with LPS *in vitro*, DHA has been shown to inhibit MHC-II expression, activation of CD86 through TLR4, expression of co-stimulatory molecules (CD40, CD80, and CD86) and inflammatory cytokine production (IL-6 and IL-12p70), therefore also inhibiting T-cell activation (24). This has also been shown *in vitro* using human DCs (25). Furthermore, *in vivo*, fish oils rich in the n-3 LCPUFAs DHA and EPA have been found to modulate the function of T-cells by suppressing signal transduction through the TCR and CD28, thereby reducing activation by DCs and proliferation of CD4^+^ T-cells (26). Proliferation of CD4^+^ T-cells has also been described to be reduced directly by incorporation of EPA, DHA and AA in the membrane in both *ex vivo* and *in vitro* studies (27, 28). Downregulation of DC-T-cell activation results in a decrease in secretion of pro-inflammatory cytokines TNF-α and IL-12 and an increase in IL-10 production and expression (22, 25, 29). N-3 LCPUFAs DHA and EPA have been described to also modulate inflammation by binding to several receptors, such as GPR120 and nuclear receptor PPARα/γ (30). Furthermore, DHA and, to a lesser extent, EPA, have been reported to prevent and reduce cow's milk and peanut allergy in mice (31) by reducing IgE, IgG1, and IgG2a levels and the generation of Treg, while lowering both Th2 and Th1 activation (32). In contrast, a 10% soybean diet, rich in-6 PUFAs, has been reported to enhance the allergic reaction to cow's milk by enhancing Th2 cell polarization and the allergic effector response (33). This could be reduced by increasing the amount of n-3 LCPUFAs in the diet, indicating that the ratio of n-3:n-6 PUFAs in the diet is important in immune system modulation (31). Therefore, unraveling the differential effects of n-3 vs. n-6 LCPUFAs or mixtures on immune cells may reveal new avenues for more specific nutritional interventions.

In the effector phase, PUFAs (n-3 alone or in varying ratios to n-6) have been shown to reduce histamine and leukotriene B4 levels when supplemented to mast cells (MCs) (34, 35). However, n-6 PUFA AA has been shown to activate intracellular ROS production, increase MC degranulation mediated by IgE, and TNF-α and PGD_2_ release (36, 37). AA is an eicosanoid precursor and can be converted via the cyclooxygenase pathway into prostaglandin H2 (PGH_2_). Both PGD_2_ and PGE_2_ are synthesized from PGH_2_, which are important in allergic symptoms by increasing vascular permeability and in maintaining allergy through the activation of Th2 cells. PGD_2_ is a prostaglandin, which is a subclass of eicosanoids, mainly secreted by MCs and one of the key molecules in the induction of food allergy symptoms (38). On the other hand, n-3 PUFAs DHA and EPA inhibit MC degranulation, and IL-4 and IL-13 secretion and PGD_2_ release by MCs (37). DHA, which like EPA, is known to compete with AA for membrane incorporation, has been shown to be negatively correlated to the AA metabolite PGE_2_ levels in human serum (39), supporting the findings that n-3 LCPUFA DHA is potentially effective in decreasing food allergy risk and symptoms in the effector phase.

Clinical trials exploring the effect of n-3 LCPUFAs during pregnancy and/or lactation on allergic outcomes (e.g., food allergy, asthma, atopy, and wheezing) are contradicting. Supplementation of fish oil starting early during pregnancy and continuing during breastfeeding was shown to reduce allergic sensitization for food proteins in the offspring (40). Lower Th2 associated cytokine levels of IL-13 were measured in the plasma of these children (41). Formula supplemented with AA and DHA was also shown to prevent allergy development in young children compared to non-supplemented formula milk (42). From epidemiological studies it is known that allergy is associated with low n-3 LCPUFAs, especially EPA and DHA, and high n-6 LCPUFAs in plasma or serum (43, 44), indicating a protective effect of n-3 LCPUFAs and the importance of aiming for an optimal balance of n-3 over n-6 LCPUFA (1:3–1:5) for immune development in neonates. However, follow up studies, often using the age group 1–5 years, report no lasting effects on sensitization prevention when n-3 LCPUFAs were supplemented during gestation (45–47). By contrast, in another study 2 years after supplementation, lower IgE levels in children whose mothers received n-3 LCPUFAs were still detected (48). In an extensive Cochrane review on the supplementation of fatty fish or n-3 PUFAs during pregnancy combining the results of eight different trials, including 3,366 woman and 3,175 children, it was concluded that the evidence for effective food sensitization and allergy prevention however is limited (49). Although a reduction in IgE-mediated allergy was observed in children between 12 and 36 months old, and reduced sensitization to egg was reported, no significant differences have been found in sensitization to cow's milk, wheat and peanut proteins. Of note, most trials (5/8) supplemented the woman with n-3 LCPUFAs prenatally only (45, 50–53), two only shortly after delivery (54, 55), whilst only one trial supplemented both pre- and post-natal (40). The latter study however showed the biggest effect on prevention of sensitization and allergic outcome in the first year of life. Hence, the timing of intervention during gestation, continuation during lactation, the dose of n-3 LCPUFA oils supplemented, the achieved levels of n-3 LCPUFA membrane incorporation and genetic pre-disposition may be determinants for possible allergy protective capacities of n-3 LCPUFA in neonates. Continuation of n-3 LCPUFA supplementation during early life maybe implicated to enforce possible beneficial effects on allergic sensitization and atopic risk during infancy (56). A summary of all LCPUFA effects in clinical, *in/ex vivo* and *in vitro* described in this review are summarized in Table 1.

**Table 1:** A summary of all PUFA related experiments and clinical trials described in this review.

**Type of cells**	**Species**	**Type of experiment**	**Type of PUFA**	**PUFA effects**	**References**
**SENSITISATION PHASE**					
DCs	Mouse	*in vitro*	DHA	Inhibition of MHC-II and co-stimulatory molecule (CD40, CD80, CD86) expression, CD86 activation and inflammatory cytokine production	(24)
DCs	Human	*in vitro*	EPA, DHA	Inhibition of co-stimulatory molecules, reduction of inflammatory cytokine production, reduced T-cell proliferation	(25)
DCs and T-cells	Mouse	*ex vivo*	EPA/DHA rich oil	PUFA exposed T-cells show reduced response to DCs	(26)
T-cells	Mouse	*ex vivo*	DHA	Reduced T-cell proliferation	(27)
T-cells	Human	*in vitro*	AA, EPA	Reduced T-cell proliferation	(28)
T-cells	Mouse	*ex vivo*	EPA/DHA rich oil	Reduced T-cell response, increased anti-inflammatory cytokine production	(29)
Treg cells	Mouse	*in vivo*	DHA rich oil	Reduced allergic response and antigen-specific IgE levels and increased Treg cells	(31, 32)
Th2 cells	Mouse	*in vivo*	N-6 rich oil	Enhanced allergic response, enhanced Th2 polarization	(33)
**EFFECTOR PHASE**					
MCs	Guinea Pig	*in vivo*	n-3/n-6	High n-3/n-6 ratio reduced MC response, histamine and leukotriene B4 production	(34)
MCs	Mouse	*in vitro*	ALA, EPA, DHA	Reduction of leukotriene B4, C4, and 5-HETE	(35)
MCs	Rat	*in vitro*	AA, EPA	AA enhanced TNFα production, AA and EPA enhanced ROS production	(36)
MCs	Human	*in vitro*	AA, EPA, DHA	AA enhanced IgE mediated degranulation, PGD_2_, and TNFα production, DHA inhibited PGD_2_, DHA, and EPA inhibited ROS, IL-4, and IL-13 production more than AA	(37)
**CLINICAL OUTCOMES**					
Clinical trial DBPCRT			Fish oil during pregnancy/breastfeeding vs. olive or soybean oil control	Reduced allergic sensitization and allergy risk, lower IL-13 plasma levels	(40, 41, 51)
Clinical trial DBPCRT			AA/DHA formula vs. plain formula	Reduced allergy risk	(42)
Clinical study Observational			EPA, DHA	Lower levels in allergic children, negatively correlated with serum IgE levels	(43, 44)
Clinical trial DBPCRT			Fish oil during pregnancy or breastfeeding	No lasting effect on sensitization or allergy risk at age 1-5 years	(45–47)
Clinical trial DBPCRT			Fish oil during pregnancy/breastfeeding vs. soybean oil control	Lower IgE levels after 2 years, reduced allergic severity	(48)
Clinical trial DBPCRT			Salmon (2 times/week) during pregnancy vs. normal diet	Lower pro-inflammatory cytokine levels in cord blood, no effect on allergy risk	(52)
Clinical trial DBPCRT			Fish oil during breastfeeding vs. olive/soybean oil control	Higher serum IFNy, no difference in food allergy development	(54, 55)

*In all clinical trials the mother received fish oil during pregnancy and/or breastfeeding and effects were measured in the children. DBPCRT, double blind placebo controlled randomized trial*.

## Combining LCPUFAs to Prevent Lipid Peroxidation

When using n-3 LCPUFAs as supplements aiming to reduce the risk of allergic sensitization in order to lower the chance of food allergy development, their high susceptibility to oxidative degradation should be carefully dealt with. Several strategies can be used to reduce lipid peroxidation of LCPUFAs. For example, Raederstorff et al. (57) stated that the intake of PUFAs should be directly linked to the vitamin E requirement. Vitamin E is a fat-soluble vitamin that has a principal role in defense against oxidant-induced membrane injury and it may have anti-inflammatory capacities as well (58). As vitamin E resides in the membrane phospholipid bilayer in cells, as do PUFAs (59), it facilitates membrane stabilization and protection against lipid peroxidation by scavenging peroxyl fatty acid radicals that will then be transferred to liquid phase anti-oxidants like vitamin C. Therefore, based on the amount of PUFAs in an average Western diet, the authors recommend a dose of vitamin E between 12 and 20 mg/day (57).

Another possible group that can help to reduce lipid peroxidation are flavonoids. Flavonoids also have anti-inflammatory effects beyond their anti-oxidant activity and can inhibit enzymes involved in the production of eicosanoids. Therefore, flavonoids have been proposed to be useful in allergy prevention (60–63). By interaction with ROS (superoxide O_2_, hydroxyl radical •OH and H_2_O_2_) and RNS (Reactive Nitrogen Species), flavonoids can terminate the chain reaction in lipid peroxidation caused by free radical formation before cell viability is seriously affected and they are able to modulate inflammatory processes (64, 65). Importantly, ROS have been shown to enhance the differentiation to Th2 cells by stimulating the production of IL-4 through the activation of STAT6 and GATA3 in a mouse model (66). The most studied flavonoid might be quercetin and its metabolites have been shown to be localized in the phospholipid bilayer (67, 68). Quercetin can be found in red wine, apples, green tea and onion and contains both anti-inflammatory and anti-oxidative properties (69). It has been reported to inhibit leukotriene B4 levels in MCs (70), reduce the gene expression of pro-inflammatory cytokines (TNF-α, IL-1β, IL-6, and IL-8) (71) and suppress inflammation in IgE-mediated intestinal epithelial cell (Caco-2) and rat basophil (RBL-2H3) activation models (72). Flavonoids have also been shown to inhibit allergic effector cells such as MCs known to contribute to allergic symptoms (73). For example, inhalation of quercetin has been shown to lower MC numbers and allergy associated cytokine levels such as IL-4, in a mouse model for allergic inflammatory lung disease (74). Therefore, it may be considered to combine LCPUFAs supplementation for food allergy prevention with flavonoids like quercetin, for its anti-oxidant as well as anti-inflammatory properties which may contribute to the effects of n-3 LCPUFA in allergy prevention. In this regard, PUFA-flavonoid hybrids or conjugates or mixed flavonoid-fish oil supplements are at the moment explored by several groups albeit not yet as a purpose to reduce allergy risk (75–77).

## Future Perspectives: Prevention and Treatment of Food Allergy

Currently, in literature there are many discrepancies between studies regarding timing and dose of the n-3 LCPUFA supplementation, as well as the described outcomes. Therefore, as mentioned previously, one strategy to more extensively study allergy prevention could be a constant supplementation of n-3 LCPUFAs to children, both during and after pregnancy (by breast feeding or formula), which will maintain adequate n-3 LCPUFA levels. Another, perhaps more interesting strategy for food allergy prevention may be to supplement pregnant women and their offspring with a combination of LCPUFAs, proper levels of vitamin E and flavonoids as additional component with anti-oxidant and anti-inflammatory properties. A balanced intake of LCPUFAs, vitamins, and flavonoids could be achieved by a simple change in diet. Mediterranean food is an example of food containing multiple bioactive dietary components and frequently proposed to be beneficial for human health, as it contains fatty fish and nuts (rich in n-3 PUFAs), olive oil (rich in oleic acids and anti-oxidants), fruits (rich in vitamins and flavonoids), and wine (rich in flavonoids) (78). Several studies have shown a positive correlation between the Mediterranean diet, consisting mainly of fish, fruits, vegetables, legumes, nuts, and cereals, during pregnancy and a reduction of allergic asthma and rhinitis (79–81). Another popular source of flavonoids and micronutrients is cocoa from the cacao tree. *In vivo*, it has been shown to have immunomodulatory properties, including suppression of IgE, TNF-α, and IL-10 levels (82, 83). Finally, a popular food supplement in the early 1900s was cod liver oil which is still being used mostly in Northern Europe countries and North America. It contains both vitamin A and D and is a sustainable source of n-3 LCPUFAs. Even though the use of cod liver oil could still be beneficial, as shown in a study on rheumatoid arthritis (84), others suggest that, because vitamin A and D are nowadays supplemented in our food, the intake could actually be harmful and positively correlated to asthma (85).

A striking observation when exploring the possibilities of LCPUFAs in food allergy is that while n-3 LCPUFAs are able to target both the sensitization and effector phase, all clinical trials and most *in vivo* studies focus only on prevention of sensitization and food allergy. Hence, studying the effects of n-3 LCPUFA supplementation either or not with additive selected nutrients having anti-oxidant and anti-inflammatory properties not only in the prevention but also for allergic symptom relief may be considered. Therefore, more studies should be conducted exploring the conditions by which n-3 LCPUFAs and other nutrients are able to reduce the risk to develop food allergy and possibly the severity of allergic symptoms.

## Author Contributions

All authors listed have made a substantial, direct and intellectual contribution to the work, and approved it for publication.

### Conflict of Interest Statement

JG is employed by Nutricia Research BV. The remaining authors declare that the research was conducted in the absence of any commercial or financial relationships that could be construed as a potential conflict of interest.
